# Muscle Carnosine Is Associated with Cardiometabolic Risk Factors in Humans

**DOI:** 10.1371/journal.pone.0138707

**Published:** 2015-10-06

**Authors:** Barbora de Courten, Timea Kurdiova, Maximilian P. J. de Courten, Vitazoslav Belan, Inge Everaert, Marek Vician, Helena Teede, Daniela Gasperikova, Giancarlo Aldini, Wim Derave, Jozef Ukropec, Barbara Ukropcova

**Affiliations:** 1 Monash Centre for Health, Research and Implementation, School of Public health and Preventive Medicine, Melbourne, Australia; 2 Institute of Experimental Endocrinology, Slovak Academy of Sciences, Bratislava, Slovakia; 3 Centre for Chronic Disease, Victoria University, Melbourne, Australia; 4 Department of Radiology, University Hospital Bratislava, Comenius University, Bratislava, Slovakia; 5 Department of Movement and Sport Sciences, Ghent University, Belgium; 6 Surgery Department, Slovak Medical University, Bratislava, Slovakia; 7 Department of Pharmaceutical Sciences, Università degli Studi di Milano, Milano, Italy; 8 Faculty of Medicine, Comenius University, Bratislava, Slovakia; INSERM/UMR 1048, FRANCE

## Abstract

**Background:**

Carnosine is a naturally present dipeptide abundant in skeletal muscle and an over-the counter food additive. Animal data suggest a role of carnosine supplementation in the prevention and treatment of obesity, insulin resistance, type 2 diabetes and cardiovascular disease but only limited human data exists.

**Methods and Results:**

Samples of vastus lateralis muscle were obtained by needle biopsy. We measured muscle carnosine levels (high-performance liquid chromatography), % body fat (bioimpedance), abdominal subcutaneous and visceral adiposity (magnetic resonance imaging), insulin sensitivity (euglycaemic hyperinsulinemic clamp), resting energy expenditure (REE, indirect calorimetry), free-living ambulatory physical activity (accelerometers) and lipid profile in 36 sedentary non-vegetarian middle aged men (45±7 years) with varying degrees of adiposity and glucose tolerance. Muscle carnosine content was positively related to % body fat (r = 0.35, p = 0.04) and subcutaneous (r = 0.38, p = 0.02) but not visceral fat (r = 0.17, p = 0.33). Muscle carnosine content was inversely associated with insulin sensitivity (r = -0.44, p = 0.008), REE (r = -0.58, p<0.001) and HDL-cholesterol levels (r = -0.34, p = 0.048). Insulin sensitivity and physical activity were the best predictors of muscle carnosine content after adjustment for adiposity.

**Conclusion:**

Our data shows that higher carnosine content in human skeletal muscle is positively associated with insulin resistance and fasting metabolic preference for glucose. Moreover, it is negatively associated with HDL-cholesterol and basal energy expenditure. Intervention studies targeting insulin resistance, metabolic and cardiovascular disease risk factors are necessary to evaluate its putative role in the prevention and management of type 2 diabetes and cardiovascular disease.

## Introduction

Type 2 diabetes is a global health problem [1]. This is largely due to increasing rates of obesity underpinned by obesogenic lifestyle. The increasing prevalence of obesity and type 2 diabetes contributes to high morbidity and mortality from diabetes complications and cardiovascular diseases with considerable associated health care costs [1,2].

Carnosine (β-alanyl-L-histidine) is a dipeptide present in mammalian tissues particularly abundant in skeletal muscle, heart muscle and central nervous system. It is available as an over-the-counter food additive and appears safe in human trials [3–8]. In animal studies, carnosine has been shown to suppress many biochemical processes that accompany aging and age related chronic diseases such as obesity, type 2 diabetes and diabetes complications, cardiovascular diseases, cancer and dementia. The mechanisms proposed have been a decrease in chronic low-grade inflammation [9,10], oxidative stress and advanced glycation endproducts [(AGEs)/advanced lipoxidation end products (ALEs) formation [11–15]. There is evidence that both chronic low-grade inflammation [9,10,16] as well as AGEs play an important role in pathophysiology of type 2 diabetes [17,18]. An increasing number of animal studies have suggested a role for carnosine supplementation in the prevention and treatment of type 2 diabetes and its complications as well as cardiovascular risk factors (adverse lipid profile and hypertension) and disease [19,20] [13–15,21] [22–31].

Beneficial effects of carnosine supplementation in humans have been shown in small clinical trials in exercise physiology, psychology, psychiatry and recently in heart failure [3–8,32]. Only two cross-sectional studies on muscle carnosine content are available in diabetes. One demonstrated increased muscle carnosine content in drug naïve patients with type 2 diabetes but not type 1 diabetes compared to controls [33]. Another study showed that muscle carnosine content is lower in patients with type 2 diabetes requiring anti-diabetic therapy compared to healthy controls [34]. We have recently shown that higher muscle carnosine was associated with progressive impairment of glucose tolerance, with the highest levels found in individuals with type 2 diabetes [35]. Given the compelling animal data on benefits of carnosine in metabolic as well as cardiovascular disease and risk factors and the limited human data in this area of research, we aimed to explore the relationships between human muscle carnosine content and anthropometry, energy expenditure, insulin resistance and daily free-living physical activity as well as cardiovascular risk factors.

## Materials and Methods

### The study population

Thirty-six sedentary non-vegetarian, non-smoking, middle-age sedentary males were recruited (lean, obese, prediabetic and individuals with newly diagnosed yet untreated type 2 diabetes, n = 9 per group). Study population is identical to Stegen et al [35] and represents a subcohort of a population characterized in detail in [36]. Volunteers underwent a rigorous protocol employing measures of obesity and insulin resistance, glucose tolerance, lipid profile, blood pressure, daily free living ambulatory activity, basal energy expenditure, fasting state energy substrate preference, metabolic flexibility as well as an assessment of muscle carnosine and circulating carnosinase 1 content. Participants had neither clinical nor laboratory signs of acute or chronic infection and did not take any medication or illicit drugs at the time of the study.

The protocol was approved by the Ethics Committee of the University Hospital Bratislava, Comenius University Bratislava and the Ethics Committee of the Bratislava Region Office (Permission Number CAR2010/33) and it conforms to the ethical guidelines of the Helsinki declaration. The participants provided both verbal and written consent and ethics committee approved the consent procedure.

### Recruitment and metabolic examinations

All volunteers were recruited from the community by the Institute of Experimental Endocrinology, Slovak Academy of Sciences, Bratislava, Slovakia between January and March 2010. Characteristics of the entire study population were published in [36]. After recruitment, volunteers underwent medical screening, including medical history & food preference questionnaire [37], physical examination and basic laboratory tests including fasting plasma lipid levels and liver function tests, an anthropometric assessment and an oral glucose tolerance test (OGTT). The study protocol was performed on two days that were separated by a 2-day interval: Day 1 consisted of the euglycemic hyperinsulinemic clamp and indirect calorimetry, Day 2 comprised skeletal muscle biopsy, OGTT and MRI.

Prior to metabolic testing, participants were asked to abstain from strenuous exercise and caffeine for 3 days. All metabolic testing was performed after a 12-h overnight fast. Analyses of circulating carnosinase and muscle carnosine levels were performed retrospectively in stored muscle samples using available biological material from the completed study [36].

### Anthropometric measurements

Body weight and height were measured and used to calculate body mass index (BMI) (kg.m^−2^). Waist circumference was measured at the midpoint between the lower border of the rib cage and the iliac crest. Quadrupedal **bioelectric impedance** was used to evaluate total fat and to estimate lean body mass (Omron BF511, Omron, Japan).

**Abdominal fat distribution** was measured by magnetic resonance imaging (MRI) using gradient recalled echo (GRE) sequence, TR: 134ms, TE: 2.38/5.24ms on 1.5 T Magnetom Symphony MRI scanner (Siemens, Germany). The area of visceral and subcutaneous abdominal adipose tissue depots was evaluated semi/automatically using the Siemens Syngo User interface (Siemens, Germany) by averaging the area of 5 consecutive slices (1 cm apart) centred between the L4 and L5 vertebrae.

### Physical activity

**Daily free-living ambulatory activity** parameters such as activity related energy expenditure, total daily ambulatory activity, number of steps per hour (when used for more than 12 daylight hours) as well as intensity of ambulatory activity were assessed by accelerometers (Lifecorder Plus, Kenz, USA) during three consecutive working days. Medium and high intensity ambulatory activity was defined as an activity with the energy requirements exceeding 3-times the resting energy expenditure (>3 MET).

### Metabolic studies

**Two-hour 75-g oral glucose tolerance test** was performed after a 12-h overnight fast and glucose tolerance was determined according to criteria from American diabetes association from 2007. **Insulin sensitivity** was assessed by hyperinsulinemic-euglycemic clamp. In brief, two intravenous cannulas were inserted into antecubital veins of both arms: first for administration of insulin, 20% glucose with KCl and second for frequent blood sampling / glycemia detection (Super GL2 -SN1440 analyzer (Dr. Muller Geratebau, Germany). A primed (80mU.m^-2^.min^−1^) continuous (40mU.m^-2^.min^−1^) insulin (Actrapid 100 IU/ml, Novo Nordisk, Denmark) infusion was used to achieve hyperinsulinemia. Blood glucose was measured in 5-minute intervals and maintained at euglycemia (5 mmol/l+/-0.5mmol/l) using variable infusion rate of 20% glucose. The whole body insulin sensitivity (M-value) was calculated from the steady state plasma glucose infusion rate required to maintain euglycemia, expressed per kg body weight per minute (M-value) and normalized to the average steady state insulinemia (μU/ml) (M-value/Insulin) of four samples taken during the last 60 minutes of the 150 minute lasting clamp. Steady state insulinemia averaged at 58 μU/mL (range 54–63 μU/mL) and did not differ across the groups.

**Resting energy expenditure (REE) and metabolic substrate preference** (respiratory quotient, RQ) were measured after an overnight fast with the aid of indirect calorimetry. Thirty minute breath-by-breath measurement was initiated after the 30-minute bed rest at thermal comfort conditions with the Ergostik (Geratherm Respiratory, Germany). Delta RQ, the parameter of metabolic substrate preference, was calculated as ΔRQ = steady state RQ (EHC)–fasting RQ. Resting energy expenditure was normalized for the variability in the exhaled minute volume.

### Muscle biopsy

Muscle biopsies of *vastus lateralis* muscle were performed on a separate day using standard aseptic technique and local anaesthesia in the fasted state. In brief, prior to a percutaneous muscle biopsy of *vastus lateralis* muscle, a scalpel blade was used to make skin incision and to cut the fascia. A side cutting muscle biopsy (Bergstrom) needle was passed through the incision to obtain approximately 100 mg of muscle tissue (aspiration). The muscle samples were immediately processed, frozen and stored in liquid nitrogen.

### Blood samples analysis

Blood samples were drawn using standard phlebotomy techniques. The tubes were centrifuged at (1,500 x g, 20 min, 4°C), and the serum stored at -20°C (glycemia, lipid profile, insulinemia) or -80°C (carnosinase 1) until analyses. During the clamp, blood glucose was immediately measured with Super GL2 -SN1440 analyzer (Dr Muler Geratebau, GmbH, Germany). Serum glucose was later reanalyzed at the certified laboratory using glucose-hexokinase 3 kit (Siemens health care diagnostics USA). Insulin was determined with IRMA (Immunotech, France), total cholesterol, HDL-cholesterol and triglycerides with diagnostic kits from Roche (Germany). Friedewald formula was used to estimate LDL-Cholesterol & atherogenic index was calculated with the formula (Total cholesterol-HDL-cholesterol)/HDL-cholesterol [38]. Free fatty acids were measured by a colorimetric assay kit (Randox, UK) and high sensitivity CRP with an immunoturbidimetric method (Randox, UK).

### Muscle carnosine and carnosinase measurements

Skeletal muscle carnosine levels were quantified by means of reversed-phase high-performance liquid chromatography (HPLC) as previously described [35].

Serum carnosinase concentrations were determined by a sandwich ELISA (enzyme-linked immunosorbent assay) developed by Adelmann [39] as previously described [36].

## Statistical Analysis

Statistical analyses were performed using SAS Jump Statistics Software (SAS, USA). Results are given as mean+/-SEM (unless indicated otherwise). Power analysis for the overall study was based on our preliminary data on interaction between glucose intolerance and intramyocellular lipid content which showed that 15% change could be revealed with the power of 80% at the significance level of 0.05 by using at least 18 individuals per group. The current analyses were not based on a power calculation using human muscle carnosine data as they were unavailable at that moment.

The relationship between muscle carnosine content, anthropometric and metabolic variables were examined by calculating Pearson correlation coefficients. Multiple linear regression models were used to examine the relationships after adjusting for covariates as well as explore potential interactions. Stepwise regression was employed to determine a relative contribution of each of the variables to the model. The variables that were significant in the univariable analyses (p<0.05) or known to be associated with muscle carnosine content from existing literature were included in the multivariable analyses. ANOVA with Tukey *post hoc* test was used to compare clinical chatractristics in 4 groups of patients (Table 1). Statistical significance was assumed when p<0.05.

**Table 1:** Characteristics of the study population.

	Lean (n = 9)	Obese (n = 9)	Pre-T2D(n = 9)	T2D (n = 9)
**Age** (years)*	47±2^a^	44±2^a^	43±3^a^	46±2^a^
**Body mass index** (kg/m2)*	24.5±0.4^a^	29.4±0.8^b^	32.0±0.7^b^	30.7±0.9^b^
**Waist circumference** (cm)*	88.2±2.4^a^	107.0±3.9^b^	111.8±2.3^b^	106.5±2.6^b^
**Body fat** (%)*	20.5±1.4^a^	28.9±2.2^b^	31.4±0.7^b^	30.3±1.3^b^
**Lean body mass** (kg)	61.8	68.3	70.6	69.0
**Subcutaneous adipose tissue** (cm2)*	149±13^a^	263±31^b^	364±30^c^	365±44^c^
**Fasting glycemia** (mmol/L)*	4.6±0.1^a^	4.9±0.1^a^	5.5±0.2^a^	8.9±1.1^b^
**2-hour glycemia** (mmol/L)*	5.51±0.40^a^	4.98±0.71^a^	8.62±0.35^b^	14.41±1.22^c^
**Fasting insulin** (μU/mL)*	2.78±1.26^a^	8.80±1.83^b^	11.8±2.05^bc^	14.48±2.02^c^
**2-hour insulin** (μU/mL)	45.3±7.9^a^	64.7±11.8^a^	110.2±14.7^b^	47.4±2.8^a^
**Fasting C-peptide** (ng/mL)	1.02±0.13^a^	1.92±0.25^b^	2.51±0.27^bc^	3.02±0.37^c^
**2-hour C-peptide** (ng/mL)	6.9±0.7^a^	9.8±1.5^a^	13.9±1.2^b^	7.9±1.7^a^
**M-value** (mg/kgBW/min)	7.51±0.44^a^	5.16±0.62^a^	3.21±0.32^b^	2.82±0.42^b^
**M/insulin** (mg/kgBW/min per μU/mL of insulin)x1000*	147±14.6^a^	86±12.6^b^	59±8.0^b^	55±10.1^b^
**HDL-cholesterol** (mmol/l)	1.4±0.2	1.3±0.3	1.3±0.2	1.2±1.2
**Steps per hour** (12h use)^#^	706±82^a^	484±80^a^	660±106^a^	689±143^a^
**Moderate & high intensity PA** (%)	39.6±4.7^a^	30.0±6.7^a^	28.6±5.7^a^	24.7±5.8^a^
**Resting energy exp.** (kcal/24h/kgLBM)	24.7±1.3^a^	21.6±1.5^b^	20.7±0.6^b^	21.5±0.9^b^
**RQ—metabolic substrate preference**	0.84±0.02^a^	0.88±0.02^a^	0.86±0.02^a^	0.90±0.05^a^
**Delta RQ—metabolic flexibility**	0.034±0.015	-0.016±0.024	0.00±0.025	-0.085±0.033
**Serum carnosinase 1** (g/mL)	15.7±1.4^a^	19.1±2.2^ab^	19.2±0.9^b^	16.7±0.65^ab^
**Blood pressure systolic** (mmHg)	122±15	119±17	119±11	128±12^a^
**Blood pressure diastolic** (mmHg)	74±8	73±8	75±8	80±11

*data previously published in the entire cohort (37);

^#^average of 2 working days where recording lasted of >12 hours, LBM—lean body mass; RQ—respiratory quotient; delta RQ = steady state (EHC) RQ-fasting RQ. Data are shown as average +/- SEM. Diferent letters of latin alphabet (a,b,c) denote statistical significance with p<0.05 (ANOVA)

## Results

The anthropometric, metabolic, biochemical and behavioural characteristics of the study population are summarized in Table 1.

### Relationships between muscle carnosine levels and measures of obesity, energy expenditure and physical activity

**Fig 1:**
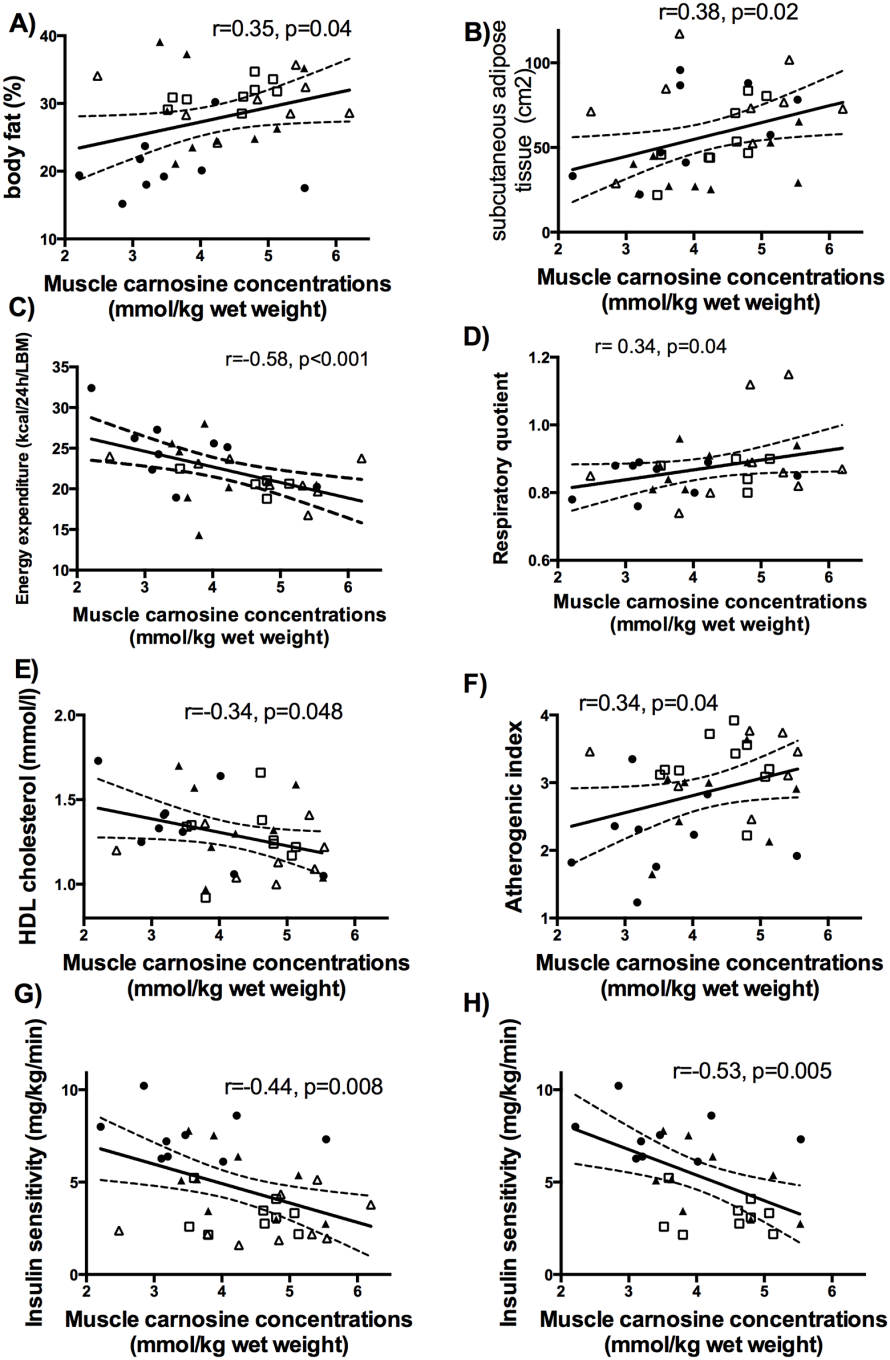
Relationship of muscle carnosine with anthropometric or metabolic parameters. • Lean, ▲Obese, □ IGT, Δ T2D, LBM—lean body mass; Respiratory quotient measured in the fasted state represents metabolic substrate preference. Insulin sensitivity—M value (mg of Glucose/kg of Body Weight/minute).

Muscle carnosine content was positively associated with BMI (r = 0.469, p = 0.007), waist circumference (r = 0.35, p = 0.03), % body fat, subcutaneous adipose tissue content (Fig 1A and 1B) but not visceral adipose tissue content (r = 0.166, p = 0.331) or lean body mass (r = 0.247, p = 0.152).

Muscle carnosine content was not correlated with the overall volume of the every day free living ambulatory activity (steps per hour, Table 1) but was inversely associated with the dynamic parameter of ambulatory activity i.e. percentage of high and moderate intensity ambulation (Table 1, association presented in [35]). There was also a negative association between muscle carnosine content and REE (Fig 1C). The relationship between muscle carnosine content and energy expenditure was independent of age and BMI (p = 0.031) and was significant after additional adjustment for level of ambulatory physical activity (p<0.05). Moreover, muscle carnosine correlated positively with metabolic substrate preference for glucose as inidicated by fasting respiratory quotient RQ (Fig 1D), and negatively with delta RQ, a parameter defining the whole body metabolic flexibility (an increase in RQ in response to the experimental euglycemic hyperinsulinemia, the state of maximal insulin-induced glucose uptake) (r = -0.421, p = 0.031).

### Relationship between muscle carnosine levels and measures of glucose tolerance, insulin sensitivity and cardiovascular risk factors

Muscle carnosine content was associated with serum fasting (r = 0.393, p = 0,018) and 2-hour glucose levels (r = 0.566, p = 0,001) as well as an area under the glycemic curve (oGTT, r = 0.554, p = 0.002). Muscle carnosine levels were also associated with 2-hour C-peptide (oGTT, r = 0.422, p = 0.023) and trend to associate was found with 2-hour insulin (oGTT, r = 0.317, p = 0.084), as well as with fasting C-peptide levels (r = 0.320, p = 0.061) and insulinemia (r = 0.310, p = 0,065). The relationship between muscle carnosine levels and C-peptide were not significant after adjustment for insulin sensitivity (M value) and adiposity (all p>0.5) possibly indicating that there is no relationship between muscle carnosine and insulin secretion independent of insulin sensitivity.

Overall, muscle carnosine content was inversely associated with insulin sensitivity (Fig 1G) and there was also a negative relationship with insulin sensitivity in non-diabetics (Fig 1H) but not in diabetics (r = 0.37, p = 0.3, p = 0.004 for interaction). Insulin sensitivity (dependent variable) was inversely associated with muscle carnosine levels (p = 0.04) after adjustment for age (p = 0.2) and % body fat (p = 0.002, r^2^ of the model = 0.49, p = 0.0004). Conversely, insulin sensitivity was a significant determinant of muscle carnosine content (p = 0.04) after adjustment for age (p = 0.7) and % body fat (p = 0.8). In the stepwise regression analysis insulin sensitivity and moderate and high levels of physical activity were the most significant determinants of muscle carnosine levels contributing to 36% of variance (p = 0.0089).

Muscle carnosine levels were negatively associated with HDL (Fig 1E) and positively with atherogenic index (Fig 1F). There were no correlations with plasma levels of total and LDL cholesterol (p = 0.7) and triglycerides (p = 0.2). There were no correlations with systolic and diastolic blood pressure (p = 0.5; p = 0.3, respectively).

### Lack of relationships of muscle carnosine with circulating carnosinase 1 content and/or dietary preference

Muscle carnosine levels were not associated with plasma levels of carnosinase 1 (p = 0.6). Plasma levels of carnosinase 1 did not differ across the groups (Table 1) and were not related to any of the anthropometric or metabolic parameters (data not shown). None of the relationships between muscle carnosine and anthropometric or metabolic parameters changed after adjustment for plasma carnosinase 1 levels (data not shown). Muscle carnosine levels were not associated with dietary preference as assessed by standardized food preference questionnair (data not shown).

## Discussion

In our study we show that human muscle carnosine content is positively associated with measures of adiposity and subcutaneous adipose tissue, respiratory quotient (parameter of metabolic substrate preference) and negatively with baseline energy expenditure. Furthermore, high muscle carnosine content was associated with insulin resistance independently of age and obesity. These results extend our previous findings that muscle carnosine content increases with progression of glucose intolerance and is negatively associated with dynamic physical activity in sedentary obese individuals [35]. Muscle carnosine was also related to HDL-cholesterol and atherogenic index.

In our previous report, we described a tendency towards an increase of muscle carnosine in obese individuals [35]. Apart from this, the relationship between muscle carnosine and obesity has not been previously described in humans. Here, we show that high muscle carnosine content is associated with different measures of anthropometry, decreased energy expenditure and increased metabolic preference for glucose, indicating relatively higher reliance on utilization of carbohydrates. Carnosine is present in brain tissue [39] and has been also shown in animals to regulate appetite [25] Intraperitoneal injection of carnosine has been shown to increase sympathetic nervous system (SNS) activity at both the peripheral and central nervous system in rodents [25,40]., a potential mechanism for the interaction between carnosine and obesity via increased energy expenditure. Possible effects on thermogenesis or lipolysis of carnosine have also been described in animal studies [25]. It is plausible that carnosine may have effects on both body weight and energy expenditure in rodents and our current findings progress this understanding with human data. Human intervention studies investigating carnosine supplementation and its effects on SNS activity, energy expenditure and body weight are necessary to see if this effect is present in humans.

A growing body of evidence from animal studies indicates a protective role of carnosine supplementation in diabetes due to its ability to affect glycaemic control but also to prevent/ameliorate diabetes complications [23,25,27,29,30]. Data from diabetic rodents indicate that supplementation of carnosine—in a dose dependent manner—reduced glucose, HbA1c, increased insulin secretion and β-cell mass [14,23,25,29,30]. Importantly, in db/db mice carnosine supplementation was able to delay the development of type 2 diabetes [29]. We recently showed that muscle carnosine content increased with worsening of glucose tolerance [35]. Here, we show that the relationship between muscle carnosine content and insulin resistance was independent of obesity. Interestingly, there was a negative relationship with insulin sensitivity in non-diabetics but no (tendency for positive) relationship in drug naïve patients with type 2 diabetes. This is consistent with previous results from 2 human studies. The first study showed increased muscle carnosine content in drug naïve patients with type 2 diabetes compared to healthy controls [33]. Whereas muscle carnosine levels were lower in patients with type 2 diabetes who have progressed to require anti-diabetic therapy compared to controls [34]Together with our data, this suggests that an increase in carnosine levels might be an adaptive mechanism counteracting increased chronic inflammation and oxidative stress, which develops in obesity, insulin resistance and diabetes. This pathophysiological model is similar to hyperinsulinemia that develops secondary to insulin resistance.

Putative mechanisms by which carnosine affects glucose metabolism, include anti-inflammatory, anti-AGEs, anti-ALEs, and anti-oxidant effects in addition to effects on the autonomic nervous system [11,13–15,25]. Other hypotheses should be considered. Among others it could be hypothesized that carnosine can act as a physiological substrate of some metabolic pathways derangement of which would result in a reduced consumption of carnosine and accumulation of carnosine. A role of carnosine as a substrate is partially supported by a very recent proteomic study demonstrating that metabolic changes in gastrocnemius muscle following serine/threonine kinase Akt1 activation include significant reductions of serine and histidine containing dipeptides (anserine and carnosine). Moreover Akt1 activation and carnosine consumption was associated to improved glucose metabolism and regression of age-related fat accumulation in old animals [41] Another possibility is that the association of muscle carnosine with measures of obesity, metabolic substrate preference and metabolic flexibility but also insulin sensitivity may be explained by fibre type composition. It is well-known that baseline carnosine levels are higher in fast-twitch compared to slow-twitch muscle fibers [42]. We [35] and others [43] have shown that muscle of type 2 diabetic or insulin resistant individuals expresses higher markers of fast oxidative type IIa fibres or fast glycolytic type IIx fibres.

With regards to cardiovascular risk factors, carnosine has been shown to improve lipid metabolism [23,44] and reduce blood pressure [19,23,45] in animal models. Specifically, carnosine has been shown to reduce lipid peroxidation [20], atherogenic ApoB containing lipoproteins (oxidized LDL and VLDL)[46], triglycerides and extracellular lipid in the plaque in rodents [47]. Here, we show that muscle carnosine is related to HDL as well as atherogenic index but not to total cholesterol, LDL and triglycerides. This could be due to relatively normal lipid profile in our study population. Further studies in dyslipidemic populations may show relationships with other lipid parameters. The anti-hypertensive effect of carnosine has been described in various mammalian species and has been attributed to direct vasorelaxing effects of carnosine [19,23] which is dose dependent [45]. Suggested mechanisms of anti-hypertensive effects of carnosine have been effects of carnosine on histamine/histidine pathway [48], nitric oxide/cGMP mechanism [45] and the effect on autonomic nervous system [19,25]. In addition, long-term effects on atherosclerosis could also be responsible for effects of carnosine on blood pressure. In our study, we did not see a relationship between muscle carnosine content and blood pressure. This was likely due to relatively small range of blood pressure and young study population studied as well as relatively small samples size, with larger studies needed. A promising beneficial effect of carnosine in humans are highlighted by a recent intervention study carried out in patients with chronic heart failure [32]A daily dose of 500 mg of carnosine for 6 months was associated to beneficial effects on exercise performance and quality of life [32].

Here, we report no relationship between muscle carnosine content and carnosinase in circulation. This is in agreement with our previous studies [3,49].

Limitations: Firstly, the cross-sectional nature of the study cannot delineate the cause-and-effect relationships between muscle carnosine and insulin resistance. Secondly, the sample size is small, and, hence, the results should be interpreted with caution. However, the strength of the correlations and their robustness in various adjusted models and use of gold standard methods for assessment of insulin resistance attests to the validity of our findings. Thirdly, it should be noted that metabolism of carnosine in humans differs from rodents because of presence of serum carnosinase 1 which quickly hydrolyses carnosine into its amino acids. However, clinical effects of carnosine have been described with chronic supplementation hence it is plausible that chronic supplementation might saturate carnosinase resulting in increased circulating carnosine levels [3–8,49]

## Conclusions

In conclusion, we have demonstrated that muscle carnosine content is linked to obesity and insulin resistance, energy expenditure, dynamic physical activity, respiratory quotient as well as HDL and atherogenic index. Intervention studies with supplementation of carnosine targeting obesity, insulin resistance and cardiovascular risk factors in individuals at risk of and with type 2 diabetes are necessary to investigate the putative role of carnosine in prevention and management of type 2 diabetes and cardiovascular disease.

## Abbreviations

AUC: area under the curve
BMI: body mass index
MRI: magnetic resonance imaging
AGE: advanced glycation endproducts
OGTT: oral glucose tolerance test
RQ: respiratory quotient
REE: resting energy expenditure
